# A time course of prosodic modulation in phonological inferencing: The case of Korean post-obstruent tensing

**DOI:** 10.1371/journal.pone.0202912

**Published:** 2018-08-27

**Authors:** Sahyang Kim, Holger Mitterer, Taehong Cho

**Affiliations:** 1 Department of English Education, Hongik University, Seoul, Korea; 2 Department of Cognitive Science, University of Malta, Msida, Malta; 3 Hanyang Institute for Phonetics and Cognitive Sciences of Language, Department of English Language and Literature, Hanyang University, Seoul, Korea; Stony Brook University, UNITED STATES

## Abstract

Application of a phonological rule is often conditioned by prosodic structure, which may create a potential perceptual ambiguity, calling for phonological inferencing. Three eye-tracking experiments were conducted to examine how spoken word recognition may be modulated by the interaction between the prosodically-conditioned rule application and phonological inferencing. The rule examined was post-obstruent tensing (POT) in Korean, which changes a lax consonant into a tense after an obstruent only within a prosodic domain of Accentual Phrase (AP). Results of Experiments 1 and 2 revealed that, upon hearing a derived tense form, listeners indeed recovered its underlying (lax) form. The phonological inferencing effect, however, was observed only in the absence of its tense competitor which was acoustically matched with the auditory input. In Experiment 3, a prosodic cue to an AP boundary (which blocks POT) was created before the target using an F0 cue alone (i.e., without any temporal cues), and the phonological inferencing effect disappeared. This supports the view that phonological inferencing is modulated by listeners’ online computation of prosodic structure (rather than through a low-level temporal normalization). Further analyses of the time course of eye movement suggested that the prosodic modulation effect occurred relatively later in the lexical processing. This implies that speech processing involves segmental processing in conjunction with prosodic structural analysis, and calls for further research on how prosodic information is processed along with segmental information in language-specific vs. universally applicable ways.

## Introduction

In spoken language, segments are always produced in an organized way within a prosodic structure of a given utterance, and phonetic realization of a segment is often determined by where in the prosodic structure it occurs, leading to position-specific phonetic and/or phonological variation [1–3]. For example, voiceless stops are produced with different voice onset times in different prosodic positions in a gradient fashion, showing prosodically conditioned ‘phonetic’ (gradient) variation [4–9]. Segments may also undergo ‘phonological’ variation largely in a categorical fashion (e.g., assimilation of a segment to phonological properties of neighboring segments) whose application may be constrained by prosodic structure [10–12]. The lack of invariance caused by the interplay between segmental and prosodic-structural levels in speech production implies that listeners should be able to modulate their perception of segments by taking into account phonetic and phonological variation in reference to the prosodic structure in which segments occur.

Speech perception studies have indeed provided evidence of prosodically conditioned modulation of speech perception [13–16]. For example, consider a phonetic form that is ambiguous between voiced (with a short-lag VOT) and voiceless (with a long-lag VOT) categories along the VOT continuum in English. This form is likely to be perceived as a voiceless stop when presented in a prosodic context in which a voiceless stop is produced with a relatively shorter VOT (e.g., phrase-medial position). However, the same phonetic form is likely to be perceived as a voiced stop when presented in a prosodic context (e.g., phrase-initial position) in which a voiceless stop is produced with a relatively longer VOT [14, 16]. This implies that listeners compute prosodic structure and modulate perception of segments by making reference to the granularity of segmental variation that arises with prosodic structure that is being computed online in speech processing (e.g., [13, 17, 18]).

The present study continues to build on the growing body of literature concerning the prosodically conditioned modulation of speech perception. Specifically, it is aimed at addressing how and when listeners process segmental variation that arises due to a prosodically conditioned phonological rule. To this end, we employ an eye-tracking paradigm to examine the time course of prosodic modulation of speech perception, investigating a case of the post-obstruent tensing rule in Seoul Korean (i.e., a lax (lenis) stop becomes tense (fortis) after an obstruent). The application of this rule is known to be constrained by prosodic structure, as we will explain in detail below.

Phonological variation occurs as a consequence of a legitimate phonological rule application. Due to place assimilation, for example, /t/ may sound like [p] as in the example of “right([p]) berry” [19]. This kind of variation often causes a temporary ambiguity because “right([p]) berry” may sound like “ripe berry” [19, 20]. When listeners encounter such an ambiguity, they rely on the preserved acoustic cues in the signal to recover the underlying form when the rule application results in incomplete neutralization [19, 21]. Even when the neutralization is complete (with no residual phonetic cues), listeners can still *infer* and recover the underlying form based on the context that licenses the phonological change, which has been discussed under the rubric of the *phonological inference account* [22–24]. Here the term *phonological inference* refers to a phonological process by which the listener may activate the lexical representation of a phonologically variable surface form by reversing the phonological rule.

A further complication to the problem arises because the application of a phonological rule in a spoken utterance is often modulated by the prosodic structure of the utterance. A rule may be applied gradiently as a function of the degree of boundary strength. For example, [25] reported relatively more frequent deletion of coronal stops in the word-final position in English when the following pause was shorter (possibly indicating a smaller prosodic boundary) than longer (indicating a larger prosodic boundary). Similarly, a higher degree of voicing assimilation (e.g., a voiced segment becomes voiceless after a voiceless segment) was observed at a smaller prosodic boundary (e.g., word) than at a higher boundary (e.g., prosodic phrase) in German [11]. In other cases, however, the rule application occurs categorically such that a rule applies within, but not across, a certain prosodic boundary. For example, a well-known sandhi phenomenon of *doubling* a word-initial consonant in Italian (often referred to as *Raddoppiamento Sintattico* or *Syntactic Gemination*) [1] applies categorically only within a certain prosodic domain, and a flapping rule in English (i.e., /t/ or /d/ becomes a flap intervocalically when the preceding vowel is stressed) is blocked across a certain prosodic boundary [1] (cf. [26]). Likewise, the post-obstruent tensification rule in Korean (i.e., a lax stop becomes tense after an obstruent), as briefly mentioned above as a test case of the present study, applies only when the segment occurs within a certain prosodic domain (i.e., Accentual Phrase [10, 27]; see below for more detail).

When encountering such prosodically conditioned phonological variation, listeners may have to accomplish a dual task. They have to resolve a temporary lexical ambiguity likely caused by the rule-induced phonological alternation while computing the prosodic structure, determining whether or not the phonological rule is applied given the prosodic environment. [15] has shown that listeners indeed consider prosodic structure when dealing with phonological variation, which was due, in this case, to the devoicing of phonologically voiced fricatives caused by a preceding unvoiced obstruent (e.g., in the phrase *hat Wälder*, Engl. “has forests,” /hat ʋɛldɐ/ → [hat fɛldɐ]). German listeners were presented with devoiced fricatives in a potentially assimilating context (i.e., the [f] in [hat fɛldɐ]). Crucially, Kuzla et al. manipulated the size of the prosodic boundary (i.e., a prosodic word boundary vs. a phrase boundary) between the potentially assimilation-triggering [t] and the potentially assimilating [f]. Listeners identified a devoiced stop as phonologically voiced more often in the word boundary condition than in the phrase boundary condition. This suggests that the listeners took into account prosodically conditioned segmental variation. That is, the fricative is indeed more likely to assimilate the voicing of the preceding segment across a word boundary than across a phrase boundary. This was the mirror image of what has been found in speech production (i.e., gradiently more devoicing after a word than a phrase boundary [11]). This study therefore provided clear evidence that listeners’ processing of the phonologically altered segments is modulated by the higher-order prosodic structure, illuminating the crucial role of prosodic structure in speech perception.

To the best of our knowledge, however, there is virtually no further study available in the literature investigating how contextually conditioned phonological variation is processed in conjunction with prosodic structure that is assumed to be computed online. In particular, little is known about the time course of speech processing that would elucidate how and when the underlying representation of a phonologically altered segment is inferred in connection with a computed prosodic structure or how it would affect lexical activation. The present study is therefore the first attempt to examine in detail to what extent the underlying form of the phonologically altered surface form is activated and how and when the activation is further modulated by the prosodic boundary. Furthermore, the present study will address another important question with respect to whether it is the prosodic structure *per se* that directly influences segmental processing, or it is a low-level normalization of prosodically-driven temporal variation that underlies the perceptual modulation of segmental variation. This will be tested by examining the extent to which perception of an identical segment is modulated depending on the preceding prosodic contexts that may carry different prosodic structural information in the absence of temporal variation (see below for further discussion on this issue).

These questions are examined in a series of eye-tracking experiments concerning the time course of the integration of segmental (lexical activation of phonologically altered segments) and suprasegmental (prosodic boundary) information through the case of the Korean post-obstruent tensing rule.

Korean stops and affricates have a phonological tense (/p*, t*, k*, tΣ*/) vs. lax (/p, t, k, tΣ/) distinction (also known as the fortis-lenis distinction; see below for the acoustic details of the distinction). (Note that ‘*’ has been widely used in the literature to refer to the tense/fortis consonants in Korean as their phonetic properties are different from tense consonants that occur in other languages; the reader is directed to [28] for more information.) The lax consonants become tensified when they occur after another obstruent, and this phonological process is known as the *post-obstruent tensing* rule (POT). As illustrated in Table 1A, when an underlying lax stop /p/ (in /**p**uri/, *beak*) occurs after an obstruent (e.g., /k/ in /porasE**k**/, *purple color*), it becomes tensified [p*] on the surface. The surface form of the underlying lax stop /p/ (= [p*], Table 1A) after an obstruent is therefore phonetically the same as that of the underlying tense stop /p*/ (Table 1B). Crucially, POT is applied within an Accentual Phrase (i.e., both within a phonological word and across consecutive words that belong to a same Accentual Phrase (AP)) but not across an AP [10, 29]. Note that, in this study, we adopted the most cited model of Korean intonational phonology, which assumes a hierarchical prosodic structure that includes at least two levels of prosodic phrases above the word level—i.e., the largest prosodic domain, the Intonational Phrase (IP) and a smaller domain, Accentual Phrase (AP) [27]. An AP in Korean is a relatively small phrase defined primarily by a specific tonal pattern at the edges, including a phrase-final H tone. Thus, when a prosodic boundary (# in Table 1) is a word boundary within an AP, POT is applied; thus, the underlying lax /p/ would be produced as a tensified stop [p*]. As a result, its surface form would be the same as that of underlying tense stop /p*/, creating ambiguity. When the boundary between the tensification-triggering context and the target consonant is an AP or IP boundary, the POT rule will be blocked [10], so the lax /p/ is phonetically manifested as a lax [p] on the surface, faithful to its underlying form without creating ambiguity between the underlying lax and the tense stops.

**Table 1:** An example of the application of post-obstruent tensing rule.

	Underlying representation (before the rule application)	Surface representation (after the rule application)
a. Underlying lax stop	/porasE**k**/ # /puri/ *purple-color # beak*	*POT applies* [porasE**k**] # [p*uri]
b. Underlying tense stop	/porasE**k**/ # /p*uri/ *purple-color # root*	*POT n*.*a*. [porasE**k**] # [p*uri]

# indicates an AP-internal word boundary.

Upon hearing the phonologically altered segment (i.e., the tensified lax) within an AP in which POT is applied, listeners would face a phonetic ambiguity between the tensified lax ([p*]uri, *beak*) and the underlying tense (/p*/uri, *root*) stops. Our first research question is then to what extent the underlying form (i.e., the underlying lax) would be activated given the phonetic ambiguity. It can be predicted that, if listeners resort to phonological inferencing, consistent with findings of earlier behavioral studies (e.g., [22–24]), they would look at both the underlying lax (i.e., via phonological inference) and the underlying tense (i.e., being faithful to the acoustic signal) stops during an eye-tracking experiment, at least before the ambiguity is eventually resolved by some other contextual cue in a given utterance.

A most crucial question follows concerning how the activation of the underlying form is further modulated by prosodic structure. This is tested by the comparison between the condition where POT is applied (within an AP) and the condition where the rule is not applied (across an AP or IP boundary). As mentioned above, the time course of prosodic influence on segmental decision has not yet been examined in detail by empirical studies, although there have been some claims based on which we could make some predictions. It has been claimed in neuro-cognitive models of processing, as discussed in [30], that segmental and suprasegmental information is separately processed in different temporal scales, and that the two levels of information are combined later for optimal lexical access. Similarly, in psycholinguistics, a proposal postulates a mechanism that analyzes prosodic structure, which is independent of, yet is computed in parallel to, segmental analysis process [13]. This mechanism, called a Prosody Analyzer, is responsible for locating prosodic boundaries (i.e., prosodic word and phrase boundaries), and the extracted boundary information is used to modulate the lexical competition process. These proposals taken together enable us to consider the possibility that prosodic modulation in phonological inferencing would be observed independently from segmental decision, occurring relatively late in the time course of the lexical activation process, which, if it exists, could be directly observed in the present study by way of the listeners’ saccades.

By comparing the lexical activation process within vs. across a prosodic phrase boundary, the current study also enables us to address another important question regarding the nature of perceptual modulation of segmental variation that arises with prosodic structure. Previous studies have shown that listeners’ segmental decisions are modulated by prosodic structure (e.g., [13–15]). For example, [14] reported that a categorical perception boundary for the voiced and the voiceless stops in English was shifted to the right along the VOT continuum, if the preceding context signaled an IP boundary as compared with when it signaled a Word boundary. In other words, a relatively longer VOT was needed for a percept of the voiceless stop when the preceding prosodic cues (both temporal and F0 cues) were consistent with an IP boundary. The authors attributed the perceptual shift to the listener’s computation of prosodic structure which was hypothesized to modulate speech perception. [16], however, further explored whether the prosodic boundary-induced modulation of segmental processing is indeed attributable to direct perception of higher-order prosodic structure *per se* or it is due to the paralinguistic temporal modulation by comparing the effect of speech rate and of prosodic boundary in speech categorization. Speech rate causes a global temporal modulation over an utterance and is indeed a well-known trigger for a perceptual shift in speech categorization [31]. A prosodic boundary also involves a localized temporal modulation at the right edge of the prosodic domain, which is generally known as a final lengthening [32–35], and the temporal variation as a function of prosodic boundary also causes a category shift [14]. In an effort to understand the exact nature of the perceptual shift that arises with temporal variation due to prosodic structure, [16] explored the effect of global temporal modulation due to speech rate versus that of local temporal modulation due to the prosodic boundary (on the phrase-final word) and found that the two conditions bring about different patterns of categorization. The different pattern associated with prosodic boundary, however, could not be unequivocally interpreted as a consequence of making direct reference to prosodic structure in speech perception as the difference could also have stemmed from a low-level temporal adjustment that varied only in terms of whether it was global or local.

This debate can be resolved if there exists a prosodic boundary with a prominent boundary cue that does not involve a temporal modulation. The Korean AP provides an ideal testbed for this question because it is demarcated by a language-specific edge tone (i.e., a high tone at the end of the phrase) without a substantial phrase-final lengthening [10]. If the listeners’ segmental decisions are modulated by the presence of an AP without any accompanying temporal cue, one could draw a firmer conclusion that listeners do compute the higher-order prosodic structure and modulate speech perception accordingly, rather than simply adjust their speech processing to low-level temporal variation. If not, then the effects of prosodic boundary-induced modulation in speech processing found in earlier studies could better be interpreted as a consequence of low-level temporal adjustment based on localized temporal variation at prosodic junctures.

To answer these questions, we carried out three eye-tracking experiments. Experiments 1 and 2 were devoted to testing two basic questions: 1) whether the phonological inferencing effect takes place in the tensifying, prosodic word context in which the POT rule applies; and 2) whether the potential ambiguity completely disappears in the IP boundary context in which the POT rule is expected to be blocked. In Experiment 3, it was then tested whether prosodic structure, when cued only by a spectral F0 cue (in the absence of temporal cues), would further modulate the expected inferencing effect, and if so, how and when in the speech processing it would occur.

## Experiment 1 and pretest

### 2.1 Experiment 1

In Experiment 1, we used the visual word paradigm [36] with printed words [37]. The goal of Experiment 1 was to test whether there indeed arises perceptual ambiguity due to phonological inferencing in the context that licenses the post-obstruent tensing (POT) rule and whether and how the prosodic boundary information may be used to guide the lexical activation of words with phonologically altered segments. The two critical conditions of a prosodic boundary between the POT-triggering contextual segment and the following target consonant were the Word (AP-internal) boundary and the IP boundary conditions. (As mentioned above, an IP is larger than an AP, so that POT is expected to be blocked completely across an IP boundary.) In the Word Boundary condition in which the POT rule is applied, the phonetically tense forms, whether derived or underlying, should induce some looks to the underlying lax stops if phonological inferencing takes places. In the IP boundary condition in which the POT rule is blocked, listeners are expected to perceive the phonetically tense forms exclusively as tense without resorting to phonological inferencing.

### 2.2 Pretest

The research questions and predictions of the present study are based on an assumption that the surface forms do not carry any residual perceptual cues to their underlying representations, so that we can investigate how the ambiguity created by the POT rule in Korean may be resolved by phonological inferencing in reference to prosodic structure. We therefore carried out a pretest to see whether the derived vs. the underlying tense forms are indeed perceived unequivocally as tense in isolation (i.e., when there is neither a phonological inferencing context nor a prosodic context).

In this section, we will first describe the methods for Experiment 1, followed by those for Pretest because Pretest was carried out with the stimuli used for Experiment 1. We will then report the results of Pretest as they will be crucial for understanding the results of Experiment 1.

### 2.3 Method for Experiment 1

#### 2.3.1 Participants

Twenty native speakers of standard (Seoul) Korean (8 males, 12 females, mean age = 24) participated in Experiment 1. All of them were undergraduate or graduate students living in Seoul who reported having normal hearing. They were paid for participation. The research protocol followed in this study and the written consent obtained from all participants were approved by the Hanyang University Human Subjects Committee.

#### 2.3.2 Stimuli

Twenty-four minimal pairs that differed only in terms of the word-initial obstruent (lax vs. tense) were selected for the experiment (e.g., *puri* vs. p*uri, cf. Table 1). The target consonants were distributed over different places of articulation, so that any observed effect would not be limited to a particular set of consonants: 10 of the pairs had a velar stop, six an alveolar stop, four a labial stop, and four an alveo-palatal affricate. The members of these pairs were recorded in a carrier sentence as shown in Table 2.

**Table 2:** An example carrier sentence with a target word “/puri/ *beak*”. # indicates a critical boundary between the target word and the preceding obstruent /k/. The carrier sentence varied in terms of the boundary condition, so that it could be either an IP boundary or a Word boundary.

		phonological context	boundary	target word	disambiguating shape	
Korean instruction	*Hwamyeon-eseo*	*pora-se****k***	#	***p****uri-wi*	*semo-lul*	*nureuseyo*
Gloss	screen-on	purple-color		beak-above	triangle-ACC	click
Translation	‘On the screen, click on the triangle above the purple beak.’					
**Phonological Context (color terms) before the target**: - Tensifying (with final /k/): *porasE**k*** (‘purple’), *y℘ndusE**k*** (‘light green’) - Non-tensifying (with final /**N**/): *y℘nnora**N*** (‘light yellow’), *y℘npunho**N*** (‘light pink’)						

The minimal pairs appeared in eight conditions obtained by crossing the following three factors: (1) intended (underlying) target (tense *vs*. lax), (2) preceding phonological context (the tensifying context with an obstruent /k/ vs. the non-tensifying context with a nasal /N/), and (3) prosodic boundary (Intonational Phrase, IP, boundary vs. Word, Wd, boundary). These factors were all included in a carrier sentence. For example, a target word (tense initial vs. lax initial) was located after a prosodic boundary marked by #, as shown in Table 2, and critical conditions regarding the phonological context and the prosodic boundary were further varied as follows.

**Fig 1:**
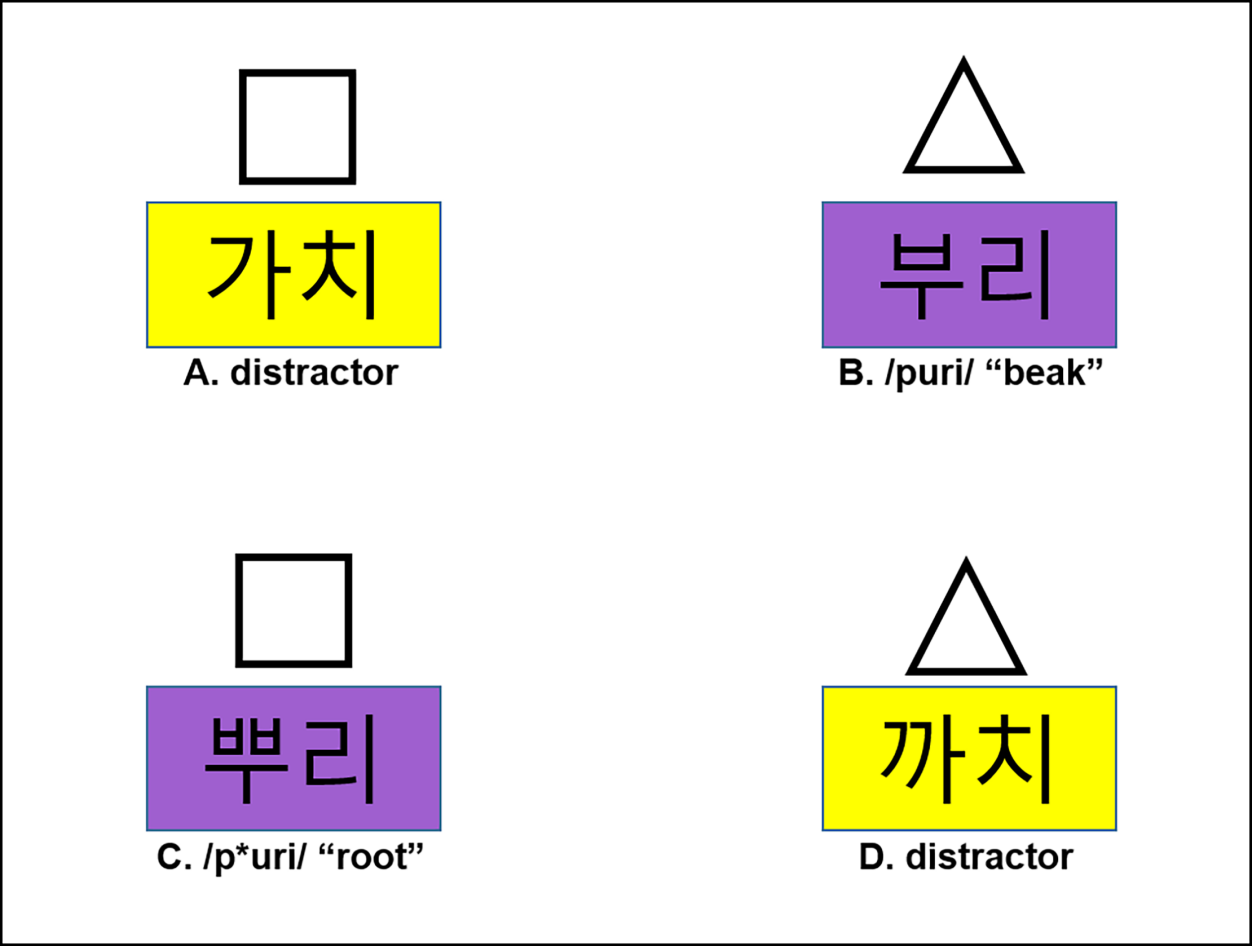
An example experimental display for Experiment 1 with gloss (A-D).

First, the phonological (tensifying vs. non-tensifying) context information was embedded in a preboundary word that was one of the four color terms. The first two color terms *purple* (/porasE**k**/) and *light green* (/y*℘*ndusE**k**/) ended with an obstruent /k/ to trigger tensification, and the other two color terms *light yellow* (/y*℘*nnora**N**/) and *light pink* (/y*℘*npunho**N**/) ended with a nasal /N/ and thus would not trigger tensification. As shown in Fig 1, these colors appeared as the background for the printed target word. Second, the boundary (#) was either an IP boundary or a Word boundary.

All target words were recorded by a female native Korean speaker in various versions of carrier sentences that differed in all the conditions discussed above (i.e., with the manipulation of the boundary (IP vs. Word) between the pre-boundary color term (ending with /k/ vs. /N/) and the post-boundary target word). It is noteworthy that the color term and the target word may be produced more frequently with a Word boundary than an IP boundary in casual speech, as noted by a reviewer, but the speaker who recorded the stimuli reported that the sentence with an IP boundary would be also a possible rendition. The two of the authors of the present study, who are experienced Korean prosodic transcribers, also concurred that the stimuli with an IP boundary sounded as natural as those with an Wd boundary.

All the recorded carrier sentences were produced with an IP boundary with a brief pause across all the stimuli for non-critical prosodic junctures of the sentence: after the first part (“**screen-on”**), which was the precursor, and before the last part, which was the post-target phrase (**“triangle/square-ACC press”**). Based on these recordings, the experimental stimuli were concatenated as follows. Several renditions of precursors and the post-target phrases were used and randomly assigned to target items. For each target word, the critical middle part (which contained a color term, a target word, and the preposition *above*) was used as recorded because the coarticulation across word boundaries made cross-splicing difficult.

For the visual displays in Experiment 1, as shown in Fig 1, the words were written in Hangul characters and placed on rectangles with four different background colors (purple, light green, light yellow, and light pink) with the size of 214 by 132 pixels. The images containing the square and the triangle were 120 by 120 pixels in size. Displays were generated from these images on a 20.5-inch screen with a resolution of 1,280 x 960 pixels. For each trial, four images with printed words were placed on the coordinates (400,300) (880,300), (400,660), and (880,660), with triangles and squares 130 pixels above these coordinates.

#### 2.3.3 Procedure

The carrier sentences were used as instructions in a visual world paradigm. Participants saw four combinations of words and shapes, and the instruction sentence was meant to guide participants with which shape to click on (which eventually disambiguated the potential ambiguity that might arise due to phonological inferencing). In the presence of an IP boundary, listeners would look at an intended target without encountering any ambiguity (thus being simply faithful to the acoustic-auditory signal of the target consonant) because POT does not apply across an IP boundary. Note that, in the Word boundary condition, given that the lax target is produced as a tensified stop due to the POT rule in the tensifying context, there might arise a temporary ambiguity if listeners resorted to phonological inferencing. The ambiguity was only temporary, however, because the combination of the instruction with visual displays in our experiments would disambiguate it (following [16, 38]). In other words, even if listeners were not sure about the intended target (lax vs. tense) due to a potential temporary ambiguity, the ambiguity was deliberately disambiguated in the carrier sentence as listeners were instructed eventually to click on a particular shape (triangle or square) above the intended target word.

To understand how this was achieved, consider the combination of the visual example display in Fig 1 and the example instruction in Table 2, which means, “*Click on the triangle above the* ***purple beak*.***”* Here, *purple* is the tensifying context (with an obstruent /k/), and *beak* is the lax target word (/puri/). When tensified due to the preceding /k/, the lax target word would be pronounced as [p*uri] (where /p/ → [p*]). The derived tense form with [p*] is also consistent with a tense competitor (/p*uri/, “root”). Given that this tense competitor is also present in the visual display (Fig 1C), the instruction is potentially ambiguous at this point. The ambiguity is, however, temporary because the instruction “click on the triangle” rules out the competitor /p*uri/ because it appears with a square (rather than a triangle) in the visual display on the screen. This disambiguating part of the instruction comes after the target word (*beak*) in accordance with the Korean word order. (See [38] for the usefulness of the use of the disambiguating shape information.) The critical display always contained a target word (in Fig 1B, /puri/ with an underlying lax target) and a competitor, which is phonetically the same in the tensifying Word context (Fig 1C, /p*uri/ with an underlying tense target). All the words on the screen were printed in Hangul (the Korean alphabet).

For Experiment 1 (and later experiments), the basic procedures were the same as those employed in [24] and [38]. Each participant was familiarized with the task with a written instruction that contained an example display. Then they performed a 9-point calibration on the Eyelink 2 head-mounted eye-tracker before performing the 216 trials of the experiment. During the experiment, the eye-tracking was checked by drift display every 10 trials and corrected if necessary. Each of the 24 items was used once in each condition that came from crossing the intended target (lax vs. tense), context (tensifying obstruent vs. non-tensifying nasal), and prosodic boundary (Intonational Phrase boundary vs. Word boundary), which yielded 192 experimental trials. In the experimental trials, the four positions on the screen were filled with the target, a competitor, and two distractors, as shown in Fig 1. When a target was a lax-initial word, the competitor was a tense-initial word, and vice versa. Two distractors came from another minimal pair with a different first segment. The target and the competitor always appeared in the same color on a given display, but in a color different from the two distractors. The target, the competitor, and the two distractors also differed in terms of shape (triangle vs. square), which was randomly determined for each trial. In a potentially ambiguous context, therefore, the target and the competitor were disambiguated by the shape, as explained earlier. The shapes associated with the target word and display positions were randomized for each participant separately. The randomization procedure was devised so that, for each participant, the 12 possible combinations of the target and competitor positions were used twice for each condition (leading to 24 trials per condition). The positions for the two distractors were then chosen at random.

In addition to 192 experimental trials, there were 24 filler trials in which the target was disambiguated either by the color or the shape. In the filler trials, the four positions on the screen were filled with two words which were presented twice. The words in these filler trials were not a minimal pair (e.g., “p*uri (*root*)” and “k*achi (*magpie*)”). The target could be disambiguated by the color because only one of the two printed words for “k*achi (*magpie*)” appeared on a specific background color (e.g., purple) mentioned in the carrier sentence, while both were accompanied by the same shape (e.g., triangle). When it was disambiguated by shape, the same two words appeared on the same background color, but only one of them appeared with the shape mentioned in the carrier sentence. The fillers were randomly interspersed in the list of 192 experimental trials. Note that the filler trials served the purpose of justifying the use of color and shape in the display, and the presence of such trials has been shown to strengthen phonological effects, possibly by focusing participant attention on sentence meaning [38].

### 2.4 Pretest

The use of the critical middle part for Experiment 1 means that the derived tense (tensified) form due to POT was used as recorded as the tensified, underlying lax target, and the underlying tense form was used as recorded as the underlying tense target. As discussed above, this experimental aspect would not entirely rule out the possibility that there may be residual acoustic phonetic cues to the underlying lax vs. the tense forms even though they appeared to be phonetically identical on the surface. We therefore investigated whether the stimuli used for Experiment 1 would have any evidence of residual phonetic cues in the tensifying context. First, we examined whether the derived tense (tensified) forms were indeed phonetically neutralized with the underlying tense forms in terms of VOT and stop closure duration, and whether a derived tense form due to POT in isolation (with no phonological or boundary context) would be identified as tense as its underlying tense counterpart.

For the acoustic analysis of the stimuli, we measured VOT and closure duration of the tensified lax stops and the underlying tense stops in 24 minimal pairs in the tensifying Wd boundary context, so that the preceding context ended with an obstruent /k/ that triggers POT.

For the perceptual (identification) test, twelve native speakers of standard (Seoul) Korean (7 males, 5 females, mean age = 24.6) participated. All of them were undergraduate or graduate students living in Seoul who reported having normal hearing. They were paid for participation. As for the stimuli, the target words were cut out of their carrier sentences and presented without a context. The listeners were instructed to choose whether what they were hearing would fit better with the lax initial word or the tense initial word. Each of the critical 24 minimal pairs (as described above) provided eight stimuli, which were obtained by crossing the target (tense *vs*. lax), context (tensifying *vs*. non-tensifying), and prosodic boundary (IP vs. Wd boundary), which eventually gave rise to 192 stimuli. The context-free auditory stimuli were presented twice each to 12 listeners with the minimal pairs written in Hangul as answer choices on the left and right of the screen (e.g., *puri* on the left, p*uri on the right). Participants were asked to press the left or right mouse button if the left or right option respectively better fit with what they heard, and their responses were coded as lax or tense.

#### 2.4.1 Results: Pretest

**Fig 2:**
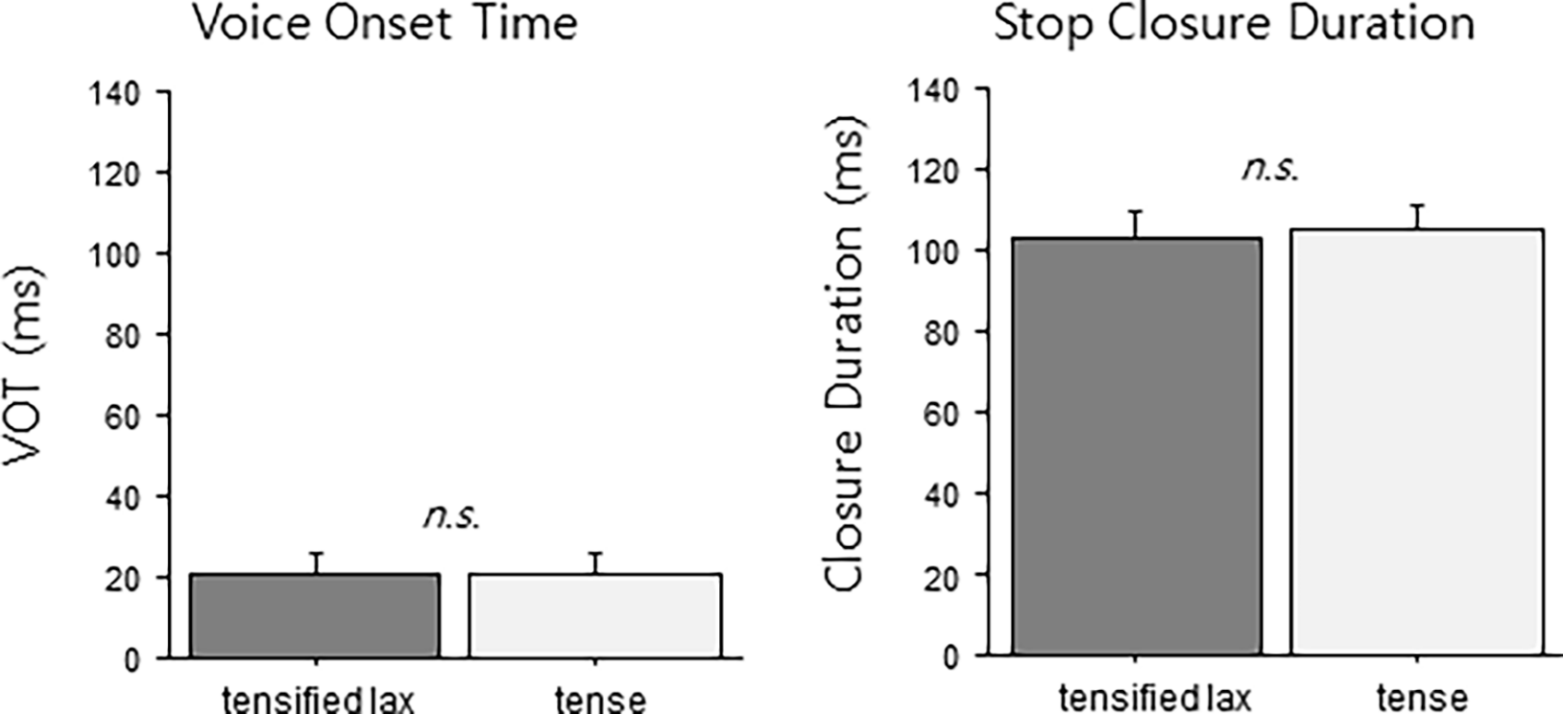
Comparisons in VOT and stop closure duration between the tensified lax stop and the tense competitor in the neutralizing (tensifying) context.

Fig 2 shows VOTs and stop closure duration for both tensified lax stops and underlying tense stops, indicating no difference between the two critical conditions. Results of paired t-tests (by item analysis) confirmed no difference (VOT, *t*(23)<1, p>0.7; closure duration, *t*(23)<1, p>0.4). To further substantiate that there is no difference between the tense and tensified productions, we ran a Bayesian t-test using the default priors implemented in the R package BayesFactor (version 0.9.12–2). This analysis leads to a Bayes Factor (BF) as a test statistic [39], which allows an estimation of how well the null hypothesis is supported by the data, with values below one third being considered good evidence for the null hypothesis. Such a BF was found both for the VOT (BF = 0.225) and closure duration (BF = 0.297). The results therefore indicated that the stimuli used in the critical tensifying context contained no residual acoustic phonetic cues to the underlying difference between the tensified lax and the underlying tense forms at least as far as VOT and closure duration were concerned.

**Fig 3:**
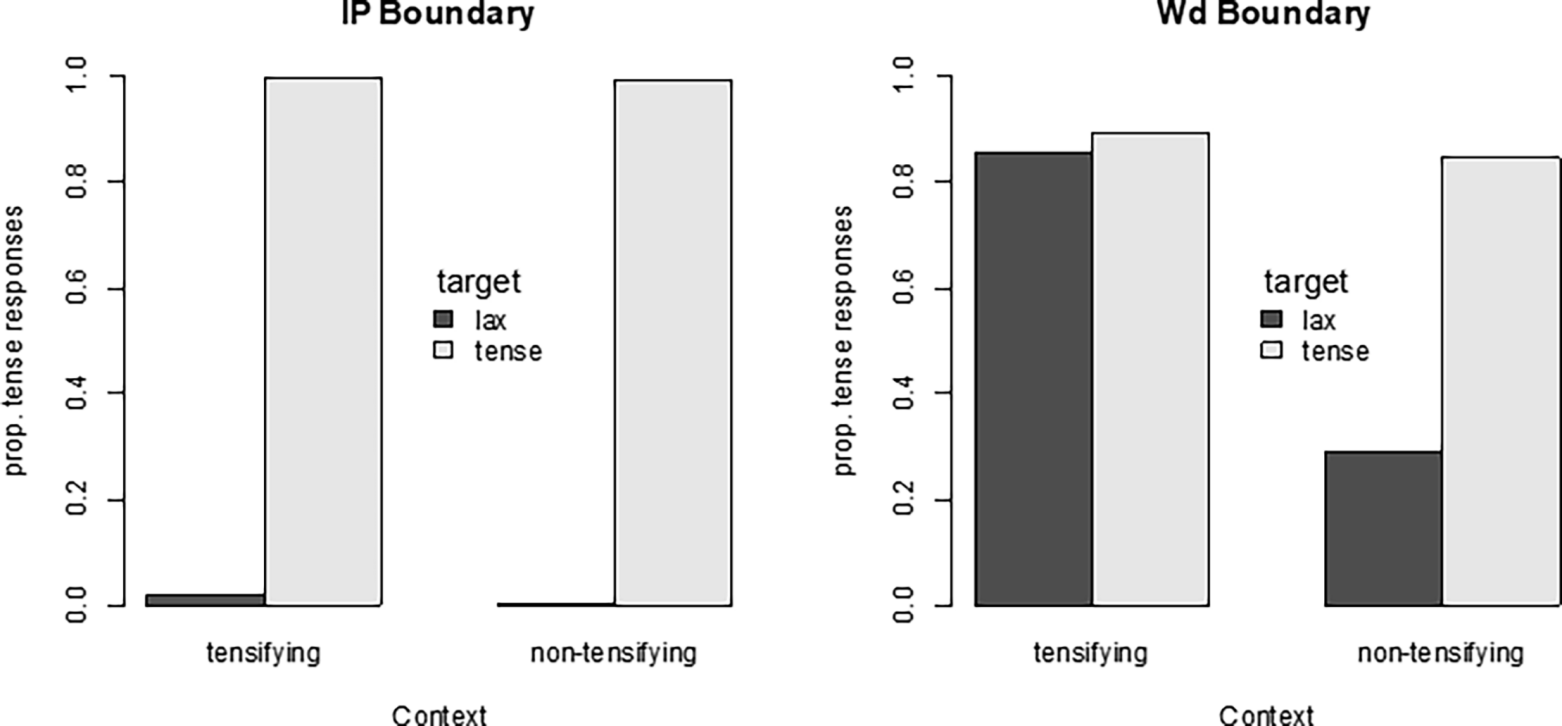
Proportion of tense responses of the target words in isolation. Target words are spliced out of each combination of intended target (lax vs. tense), context (tensifying vs. non-tensifying), and prosodic boundary (IP vs. Wd). Note that the coding of the dependent variable as tense responses (rather than “correct” responses) means that the pattern in the IP boundary condition shows near-perfect transmission of the phonological contrast.

Fig 3 shows the proportion of tense responses for each of the eight conditions. The left panels show the results of the identification of target words spliced out of the IP boundary sentences in which the POT was blocked. As can be seen in the figure, there was a near-perfect separation between lax and tense targets, which was exactly as expected because there would be clear phonetic differences between the underlying lax and the underlying tense targets when the lax stop did not undergo the POT rule. The right panels of Fig 3 show results in the Wd condition (i.e., the target words were spliced out of the Word boundary sentences). The tokens spliced from the non-tensifying context demonstrated a clear distinction between the lax and the tense targets, although the separation was not as perfectly polarized as in the IP condition. It is worth noting that the word boundary condition here means that the lax stop is produced in the middle of a phrase. In the phrase-medial (word-initial) context, the lax stop undergoes a reduction of aspiration, to be produced with a short-lag VOT or often with some degree of voicing during the closure, a phenomenon known as the lenis voicing rule (e.g., [10]). The actual initial lax stop in a word in isolation is therefore produced with more aspiration than the lax stimuli spliced from the (phrase-medial) word boundary context. The listeners would then have expected more aspiration for a canonical lax stop in isolation than the spliced lax stop stimuli that they heard in isolation in the pretest. Given that the tense stop is produced with a shorter VOT than the lax stop, the lax stop stimuli with a reduced VOT presented in isolation may have created some listener bias toward perceiving a tense stop (cf., [14,16]). Most crucially, however, in the tensifying context, participants heard both lax and tense targets overwhelmingly as tense, showing that the speaker applied tensification as expected.

Given the near-perfect separation in the IP condition, it turned out to be difficult to statistically analyze these results using generalized linear mixed-effect models with a binomial (i.e., logit-link) function with data pooled across the IP and Wd conditions. Statistical analyses hence focused on the most critical Wd condition. Here a model with target and context as contrast coded fixed factors (target: tense = -0.5, lax = 0.5; context: non-tensifying = -0.5, tensifying = 0.5) was used to predict the proportion of tense responses. Subject and target words were used as random factors, with the maximally converging random effect structure (random slopes for context over participant and item). The analysis revealed a significant interaction of target by context (B = 4.148, SE = 0.681, z = 6.090, *p* < 0.001). To understand the nature of the interaction, separate models with only targets as fixed factors were performed for tensifying and non-tensifying contexts. This revealed an effect of target in the non-tensifying context (B = -5.139, SE = 0.679, z = -7.565, *p* < 0.001) but no effect of target in the tensifying context (B = -0.532, SE = 0.456, z = -1.167, *p* > 0.2). The results therefore indicate that the stimuli with phonetically tense forms in the tensifying context (whether derived from the underlying lax or originating from the tense) do not carry any residual perceptual cues to the underlying representations.

### 2.5 Results for Experiment 1

Results showed that participants clicked on the target word as instructed by the carrier sentence. Even in the tensifying condition, 98.9% of the clicks were on the intended lax target as guided by the instruction of the carrier sentence, indicating that the listeners followed the instruction on almost all trials most of the time. Due to this ceiling effect, it was not feasible to run a statistical analysis on this aspect of the listener performance.

**Fig 4:**
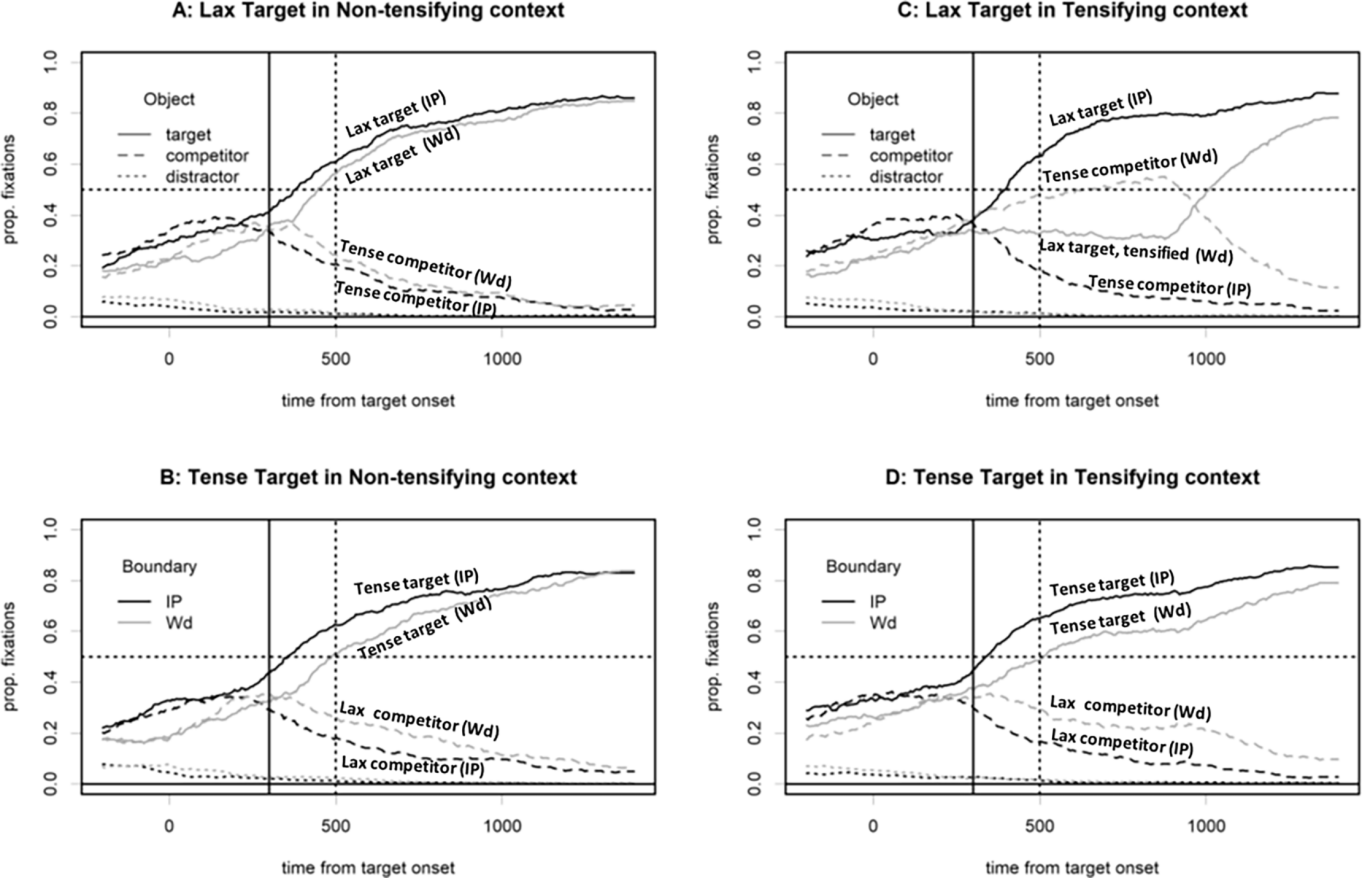
Fixations on the target, the competitor, and the distractors for each combination of intended target, context, and prosodic boundary. The line type indicates object type (target vs. competitor vs. distractor), and line darkness indicates Boundary.

Fig 4 shows the eye-tracking data for each condition, comparing effects of the prosodic boundary for each combination of the intended target and the phonological context. As expected, the data show a more efficient word recognition overall in the IP boundary (marked by darker lines in Fig 4) given that the surface phonetic forms are simply faithful to the underlying representations due to the blocking of the post-obstruent tensing rule. Regardless of the intended target and the context, there were always more looks to the target in the IP-boundary than in the Wd boundary condition. As shown in Fig 4C, however, for the critical tensifying context in the Wd boundary condition, listeners generally preferred to look at the tense competitor rather than the intended lax target upon hearing the derived tense (tensified) form of the underlying lax target. In other words, despite the presence of the tensifying context that licensed the tensification of the lax stop, no phonological inferencing effect was observed.

A linear mixed effect model was fit with the logit-transformed proportion of looks to the target in a 300-700ms time window using all three factors as contrast-coded predictors (Target: lax = 0.5, tense = -0.5, Boundary: IP = 0.5, Wd = -0.5, Context: tensifying = 0.5, non-tensifying = -0.5). Subject and item were used as random factors with all possible random slopes specified (i.e., the maximal random effects structure, see [40]), but with no correlations between random effects. The results shown in Table 3 reveal a three-way interaction. The interaction was further analyzed by testing the effects of Boundary and Target for tensifying and non-tensifying contexts separately. In the non-tensifying context (Fig 4A and 4B), there is only a significant effect of Boundary (b = 0.889 (0.189), t(16) = 4.696, *p* < 0.001, with more looks to the target in the IP-boundary condition) while the effect of Target and the interaction were only trends (Target: b = 0.294 (0.163), t(20) = 1.789 *p* = 0.087, Target x Boundary: b = -0.597 (0.323), t(48) = -1.850, *p* = 0.071). In contrast, in the tensifying context, there were not only main effects of Boundary (b = 2.042 (0.181), t(20) = 11.276, *p <* 0.001) and Target (b = -0.907 (0.249), t(20) = -3.646, *p <* 0.001), but also a significant interaction (b = 1.398 (0.396), t(20) = 3.522, *p =* 0.002). This means that the overall effect of prosodic boundary was larger for lax targets due to the tensification in the Wd boundary condition that led to more looks to the tense competitors.

**Table 3:** Results of the overall analysis of the target looks in Experiment 1.

Effect	B (SE)	T	p
Intercept	0.272 (0.313)	0.871	0.393
Boundary	1.463 (0.159)	9.220	<0.001
Context	-0.412 (0.113)	-3.661	<0.001
Target	-0.305 (0.176)	-1.731	0.096
Boundary:Context	1.157 (0.225)	5.138	<0.001
Boundary:Target	0.404 (0.243)	1.663	0.112
Context:Target	-1.204 (0.260)	-4.628	<0.001
Boundary:Context:Target	1.996 (0.450)	4.432	<0.001

These data reflect mostly the effects of tensification in the stimuli. Contrary to our prediction, there are far more looks to the competitor (i.e., underlying tense targets) than to the target for tensified lax targets in the Wd boundary condition (Fig 4C). In fact, there is no significant difference between the looks to the tense words when they function as competitors in the tensifying conditions (i.e., upon hearing the derived tense forms) and when they function as unambiguous targets in the non-tensifying conditions (upon hearing the underlying tense forms). That is, listeners seem to be faithful to the phonetic signal and do not consider the possibility of tensification, even though they are eventually instructed to click on the intended lax target after hearing the shape word, which could have induced some kind of learning effect.

We therefore analyzed whether the looks to the tense items differed between the conditions in which the targets were produced as tense on the surface, regardless of whether the phonetic tense form was derived or not, across three cases in the Word context: the derived tense form due to POT, the underlying tense form in the tensifying context, and the underlying tense form in the non-tensifying context. For the same time window (300-700ms), we compared the proportion of looks to the tense word based on the condition. If listeners were to take into account the ambiguity in the tensifying context (resorting to phonological inferencing), there should be a significant effect of condition (with three levels: tensifying-lax target, tensifying-tense target, and non-tensifying-tense target), but no such effect was observed. The proportions of looks to the tense word in the three conditions were roughly similar (lax targets in tensifying context: 39%; tense targets in non-tensifying context: 43%; tense targets in tensifying context: 46%). These looks were clearly signal driven, since the looks to the tense word were much less with a lax target in non-tensifying condition (i.e., with 4% of looks to the tense words and 58% of the looks to the lax target). A comparison of a model with the condition predictor and one without it showed no significant effect (X^2^(2) = 1.367, p > 0.2).

### 2.6 Discussion

The results revealed a clear effect of IP boundary on speech perception in connection with the domain of POT. Across an IP boundary where POT is not applied, listeners simply looked at the target as intended, consistent with the results of the pretest. Participants indeed gave more looks to intended targets (and therefore fewer to competitors) in the IP than the Wd condition. In the Wd condition where POT was applied, listeners were also faithful to the surface acoustic signal, which led to more looks to the tense competitors upon hearing the derived tense (tensified) form of the intended lax target. That is, there was no clear evidence showing that the listeners were making any phonological inference. They only reverted their look to the intended target (i.e., the lax target in the tensifying context) when the sentential context (i.e., the instruction of the carrier sentence) provided a cue for disambiguation by the shape. The results therefore allow an interpretation that participants would have treated the tensification as simply a mispronunciation and followed the instruction in the carrier sentence. This interpretation might be best explained with an analogy; consider an experiment in which you are asked to *click on the pen above the star*, and the visual display contains the written word *pan* above a star and the word *pen* above a circle. As a participant, one might simply assume that the speaker made a mistake and click on the word *pan* because it is above the object (shape) indicated in the instruction sentence. Such a simple explanation would explain the current results. This is indeed in line with what Fig 4C (for the lax target in the tensifying context) showed: Initially, listeners had a preference for the tense interpretation but reversed it upon hearing the name of the object.

However, given the evidence in the literature that listeners do make phonological inference in processing phonological variation [21–23, 41], if they indeed infer the underlying phonological form based on the phonological context especially across a Word boundary, there should have arisen some degree of activation of the lax target word upon hearing the phonetically tense form (whether derived or faithful to the underlying representation), at least relatively more strongly in the tensifying phonological context than in the non-tensifying context. But as the results of the pretest indicated, the derived tense (tensified) forms (of the underlying lax consonants) presented to the listeners in isolation were accepted as the tense category as much as the underlying tense forms. One cannot therefore rule out the possibility that phonological inferencing actually took place, but listeners, upon hearing the phonetically tense form (whether derived or underlying), simply preferred to look at the tense competitors and relied on the match between the acoustic auditory input (with the phonetic tense form) and the tense competitor available on the screen. In Experiment 2, we continued to explore this possibility with a modified experimental design.

## Experiment 2

**Fig 5:**
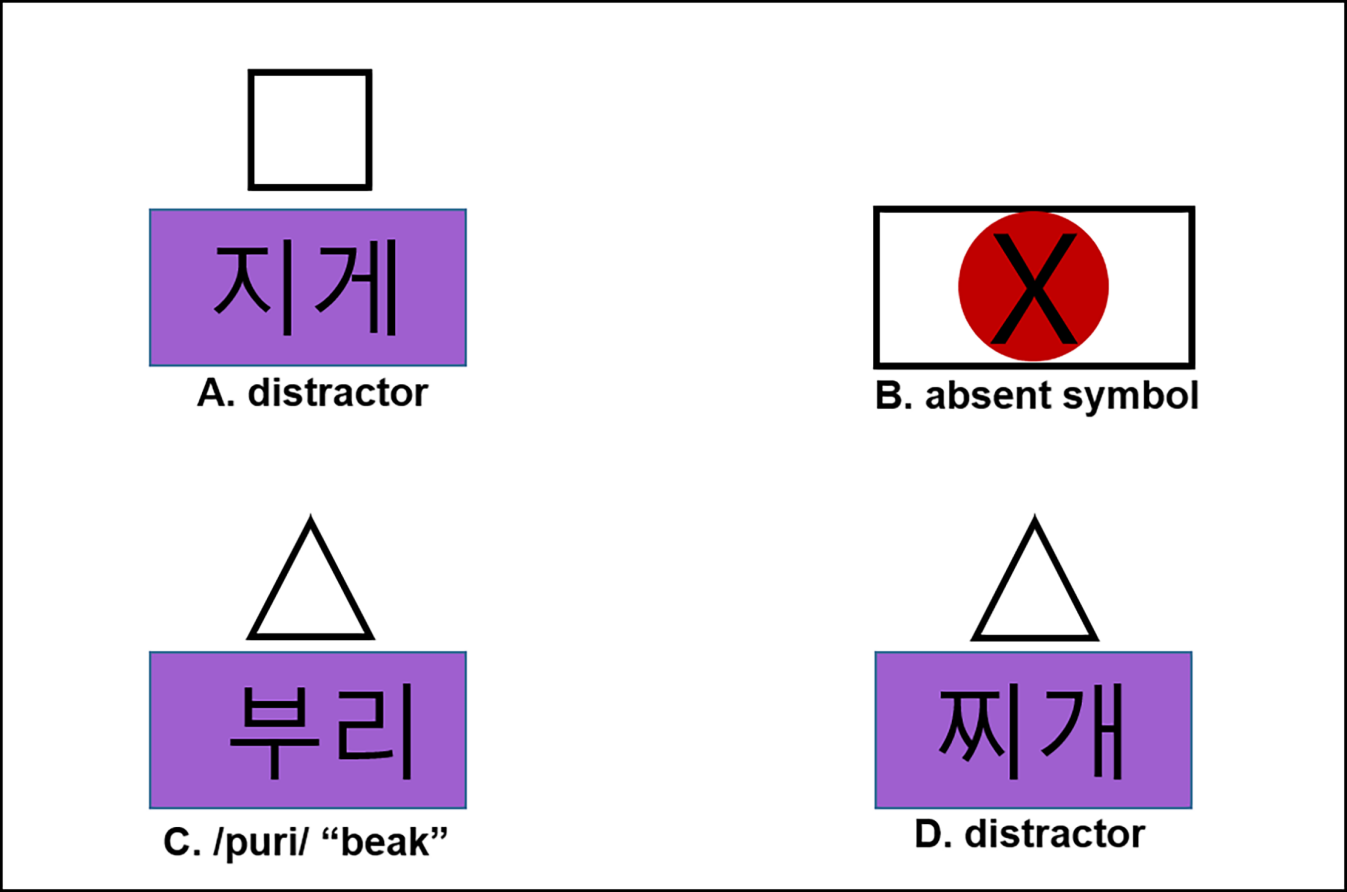
An example experimental display for Experiment 2 with gloss (A-D).

Experiment 2 tested more directly whether or not listeners would show a phonological inferencing effect in the Word boundary condition that licenses POT. In particular, Experiment 2 was designed without the tense competitor displayed on the screen in order to eliminate the listeners’ preference to look at the tense competitor on the screen, which could be simply driven by being faithful to the acoustic-auditory input that matches with the tense competitor even when some degree of phonological inferencing may have taken place. The new design leaves three positions, each with a visual word and a shape on the screen as in Experiment 1, and the fourth position filled with an “absent” (**⊗)** symbol (a red circle with a cross inside), as shown in Fig 5. Participants were asked to click on this “absent” symbol if they thought there was no word on the screen that matched the auditory stimulus. Crucially, there were always two distractors and the “absent” symbol, and the pivotal item was always a lax-initial word, as in Fig 5C, which made it possible to examine the extent to which listeners indeed looked at the lax target word upon hearing a phonetically tense form (whether derived or underlying).

Let us consider, for example, a trial in the Word boundary condition with an instruction, “*Click on the triangle above the* ***purple beak***,*”* where the final consonant in *purple* (/porasE**k**/) provides a tensifying context and *beak* (/puri/) is the target word. Again, recall that “click on the triangle” comes later in the instruction sentence according to Korean word order. Because the lax target word “puri (*beak*)” is produced in the tensifying context, listeners would hear a phonetically tensified [p*uri], which could be ambiguous with underlying “p*uri (*root*).” The display, however, contains only the lax-initial word “puri (*beak*)” as a possible target. If listeners simply go with the surface phonetic form without phonological inferencing, they would think there was no target available on the screen, clicking on the “absent “symbol (Fig 5B) because there is no tense competitor (“p*uri (*root*)”) on the display. Alternatively, if listeners indeed make a phonological inference and consider the lax target (“puri (*beak*)”) as the intended target, they would click on the actual lax target upon hearing a derived tense form in the tensifying context. The degree to which listeners click on and look at the lax target in the tensifying context compared to that in the non-tensifying context, therefore, would allow us to estimate the degree of phonological inferencing in this experimental design.

As in Experiment 1, there was a critical tensifying condition, the “/k/+lax” condition, in which listeners heard a tensified lax word with the preceding word ending on an obstruent (i.e., a tensifying context), but there was only a lax target on a display without a tense competitor. In this condition, as discussed above, if listeners retrieve the underlying representation (through phonological inferencing), they should click on the shape over the lax target word, but if they are only faithful to the surface phonetic information, they should click on the absent symbol. The other two conditions included a non-tensifying context (with /N/ before the target word); therefore, the phonetic surface form of a consonant was the same as the underlying form. In the “/N/+tense” condition, a tense word was provided with the preceding non-tensifying context in the auditory carrier sentence, so the initial consonant was produced as a tense stop, and again there was a lax target on a display without a tense competitor. In this condition, which does not license the tensing rule, listeners were supposed to click on the “absent” symbol, not the lax word. Finally, there was a “/N/+lax” condition, in which listeners heard a lax initial consonant produced as is with the preceding non-tensifying context. In this condition, they were expected to click on the lax target. It is therefore expected that the click on the lax target would be most likely observed in the non-tensifying “/N/+lax” condition and least likely observed in the “/N/+tense” condition. Crucially, if phonological inferencing is at work, more clicking on the lax target would be observed in the tensifying “/k/+lax” condition than in the non-tensifying condition upon hearing a tense form.

### 3.1 Method

#### 3.1.1 Participants

Twenty native speakers (11 males, 9 females; mean age = 24) of standard Korean participated in this study. They were undergraduate or graduate students with normal hearing and were paid for participation.

#### 3.1.2 Stimuli

The same 24 minimal pairs and the same experimental auditory stimuli as in Experiment 1 were used.

#### 3.1.3 Procedure

For each participant, the experiment consisted of 220 trials. Each experimental session started with four practice trials containing two examples with a display in which the target was absent. There were three experimental conditions (/k/+lax, /N/+lax, /N/+tense), and the 24 lax targets were presented once in each condition. As aforementioned, for these critical conditions, there was no tense competitor on a display. There were two additional conditions with lax targets presented with a tense competitor on the display, in which the lax target word was separated from the preceding context by an IP boundary or with a word boundary and a preceding non-tensifying context. In both cases, the lax target word was produced with an unaltered clear lax stop, so that there was no mismatch between the phonetic form and underlying form of the target. Four additional conditions made use of tense targets, so that the overall likelihood of the intended tense vs. lax targets was balanced. In one of these conditions, the tense targets were presented without a lax competitor so that displays with three objects occurred both with the lax and tense targets.

As in Experiment 1, the list of trials was randomized for each participant, with the constraints that the target and competitor positions were balanced within each condition for each participant. Participants were instructed to click on the shape as indicated by the sentence they heard and to click on the “absent” symbol if they thought that no matching object was present on the screen.

### 3.2 Results

Participants accepted the lax target as intended with 99.8% of the clicks on the lax target (upon hearing the lax phonetic form) in the non-tensifying “/N/+lax” condition, with 50.2% of the clicks on the lax target (upon hearing the derived tense phonetic form) in the tensifying “/k/+lax” condition, and with just 8.8% of the clicks on the lax target (upon hearing the tense phonetic form) in the “/N/+tense” condition. We used a generalized, linear mixed-effect model with a binomial linking function to analyze these data. The tensifying “/k/+lax” condition was mapped on the intercept, and two dummy coded variables were used for the non-tensifying “/N/+lax” and “/N/+tense” conditions. Participant and item were used as random effects with uncorrelated random slopes for both dummy variables. Due to convergence issues, the random slope for a full match over items was removed. The resulting model revealed significantly more clicks on the lax target in the non-tensifying “/N/+lax” condition than in the tensifying “/k/+lax” condition (b = 7.114 (1.033), z = 6.882, *p* < 0.001) and significantly more clicks on the lax in the “/k/+lax” condition than on the lax target in the “/N/+tense” condition (b = -3.35 (0.366), z = -9,110, *p* < 0.001).

**Fig 6:**
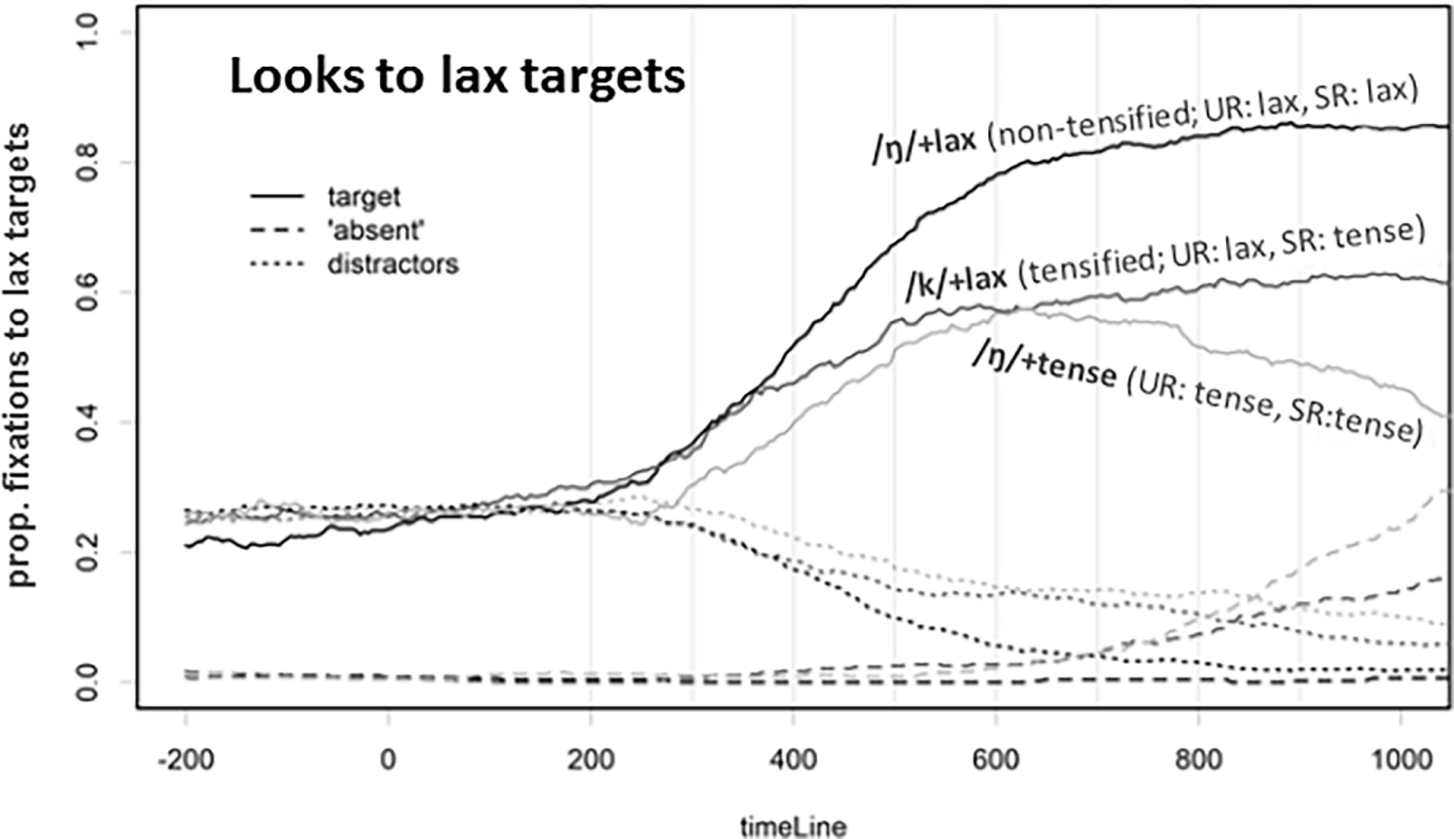
Fixations on the lax target, the “absent” symbol, and the distractors in Experiment 2. The line type indicates object type (target vs. absent vs. distractors), and the critical comparison is between /k/+lax (tensified; Underlying Representation, UR = lax; Surface Representation, SR = tense) and /ŋ/+lax (non-tensified; Underlying Representation, UR = tense; Surface Representation, SR = tense).

The eye-tracking data, as shown in Fig 6, also reveal some evidence of phonological inferencing. As shown in Fig 6, early on in the process (i.e., before 400ms), listeners look to the lax target to a similar extent in both the non-tensifying (“/N/+lax”) and the tensifying (“/k/+lax”) conditions. There is some divergence of this pattern at around 400ms in such a way that there are more looks to the lax target in the “/N/+lax” (upon hearing the phonetically lax form) than in the tensifying “/k/+lax” condition (upon hearing the phonetically tense form), while the difference between the “/k/+lax” condition and the “/N/+tense” becomes progressively smaller from 400ms onward. In later time windows (roughly from 700ms onward), as can be inferred from Fig 6, there is again a three-way separation between the conditions.

To analyze the time course, we used a moving time window analysis (see Table 4) with 100ms time windows. Fixation proportions were logit-transformed and analyzed with a linear mixed effect model with participant and item as random factors with a maximal random effects structure. These analyses, especially as summarized in the right column of Table 3, showed that, indeed, there is a significant difference between the tensifying “/k/+lax” condition and the “/N/+tense” condition shortly after hearing the word (in earlier time windows, especially from 200–400ms), which then fails to reach significance from 400–800ms, but then re-emerges from 800ms onward. That is, the phonetically similar tense form is indeed perceived differently depending on whether the preceding context licenses the tensing rule (i.e., with more looks to the lax target in the tensifying /k/+lax context).

**Table 4:** Results from the moving time window analysis from Experiment 2.

time window	/N/+lax			/N/+tense		
	b (SE)	t	p	b (SE)	t	P
200–300	-0.069 (0.174)	-0.399	0.690	-0.410 (0.174)	-2.353	0.019
300–400	0.127 (0.179)	0.709	0.479	-0.425 (0.180)	-2.366	0.018
400–500	0.613 (0.221)	2.778	0.010	-0.307 (0.183)	-1.676	0.094
500–600	1.009 (0.179)	5.640	< .001	-0.249 (0.214)	-1.160	0.251
600–700	1.426 (0.176)	8.120	< .001	-0.091 (0.213)	-0.426	0.672
700–800	1.507 (0.175)	8.636	< .001	-0.347 (0.197)	-1.766	0.082
800–900	1.553 (0.180)	8.613	< .001	-0.717 (0.195)	-3.669	0.001
900–1000	1.528 (0.218)	6.998	< .001	-0.970 (0.234)	-4.147	< .001

### 3.3 Discussion

It was found that listeners accepted lax targets more often with phonetically tense forms when they were justified in a phonological context. In the tensifying context that licenses the POT rule, they accepted lax words as intended targets even though what they heard were phonetically tense (i.e., tensified) stimuli. Furthermore, listeners gave significantly more looks to the lax target in the tensifying than in the non-tensifying context, both in the relatively earlier (between 200-400ms) and later (after 800ms) time windows. It appears that listeners initially entertain the possibility that the phonetically tensified sound heard in the tensifying context is indeed from the underlying lax through phonological inferencing, which is weakened in the middle of the processing but reinforced later. The results taken together, from proportions of both clicks and looks, unequivocally show strong evidence of phonological inferencing within the rule-triggering segmental and prosodic (Word-boundary) context. That is, the looks to the lax item in the tensifying condition was not simply triggered by the object name but it was also facilitated by the tensifying context. Were this not the case, we would have observed a similar behavior in the “/N/+lax” and “/k/+lax” conditions.

The subsequent, and most important, research question is then how and when the listeners’ phonological inferencing would be modulated by their computation of the prosodic structure. In general, a strong prosodic boundary such as an IP boundary, as used in Experiment 1, is accompanied by multiple prosodic cues in both temporal and spectral dimensions such as final lengthening and edge tones. An important issue addressed by the present study, as we discussed at its outset, is to what extent perceptual adjustment that arises with prosodic variation in the speech signal is not simply attributable to a low-level temporal modulation localized at the prosodic juncture, but instead comes about as a consequence of the direct computation of a high-order prosodic structure. In Experiment 3, we explored this issue by testing how the phonological inferencing effect observed in Experiment 2 can be further modulated by a prosodic F0 cue without any temporal cues, so that any observed effect can be more readily interpreted as a result of direct computation of a high-order prosodic structure.

## Experiment 3

In Experiment 3, we provided a prosodic context where a prosodic boundary can be perceived without temporal cues such as a pause or lengthening. As discussed in the introduction, such a context is obtained using an F0 cue associated with an AP boundary in Korean, which is not generally marked by a substantial final lengthening. We then tested whether, and if so how, the prosodic boundary manifested by an F0 boundary cue alone contributes to the prosodic modulation in the processing of phonological variation due to a phonological rule, again in this case, the POT rule in Korean. To this end, only the tone immediately before the target consonant (i.e., the end of the preboundary color term) was manipulated, so that it is consistent with a high tone that signals the end of an AP. If listeners indeed exploit the F0 cue alone and compute prosodic structure in association with an AP, they should be able to modulate their phonological inferencing depending on the computation of prosodic structure. In other words, given that POT is not applied across an AP boundary, if listeners interpret a high F0 in the preceding context as being attributable to an AP boundary, the effect of their phonological inferencing is expected to disappear. In such a case, listeners would not look to the target lax on the screen upon hearing a tense form even in the “/k/+lax” context that licenses the POT rule because the tensification is no longer justified in the presence of a phrase-final H tone.

### 4.1 Method

#### 4.1.1 Participants

Twenty native speakers (8 male, 12 female; mean age = 22.9) of standard Korean participated in this study. They were undergraduate or graduate students with normal hearing and were paid for participation.

#### 4.1.2 Stimuli and procedure

The stimuli and procedure were the same as in Experiment 2, except that the pitch contour of the preceding context word was adjusted with PSOLA to create the perception of an AP prosodic boundary without changing the temporal properties of the stimuli. To achieve that, we changed the pitch contour during the second half of the phrase containing the context word that directly precedes the target (see Table 2) with an F0 rise to 170% of the original pitch contour. (It is an empirical question to determine how much rise should be enough to create a clear percept of AP boundary. We modeled our female speaker’s production so that it showed a phrase-final boundary tone with a 170% F0 rise on average compared to the phrase-medial tone.)

### 4.2 Results

**Fig 7:**
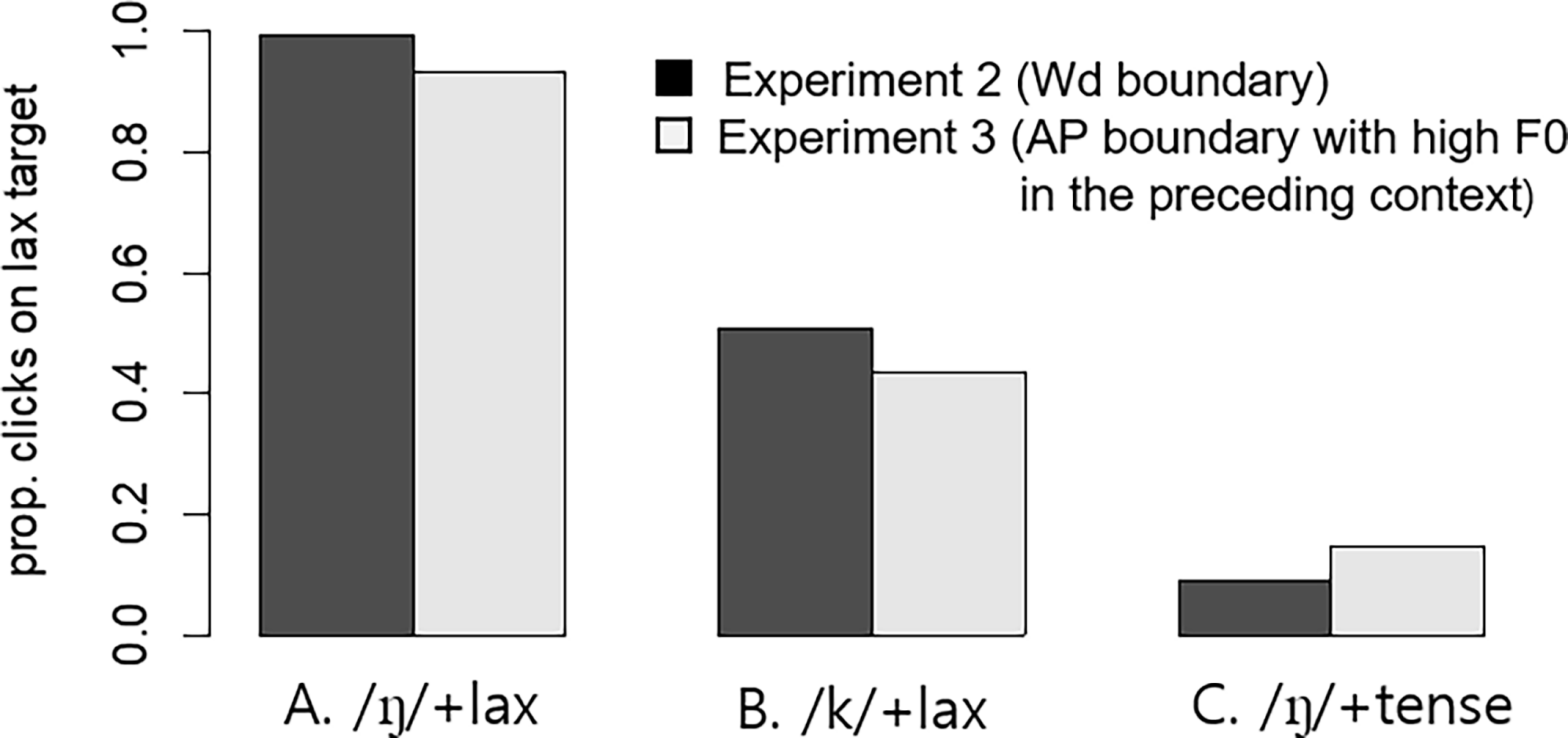
Proportions of clicks on a lax target in Experiments 2 and 3. (A) The “/N/+lax” context (a ‘lax’ phonetic form); (B) The “/k/+lax” context (a ‘derived tense’ phonetic form); (C) The “/N/+tense” context (an ‘underlying tense’ phonetic form). The auditory stimuli were embedded in the Word boundary context with no F0 rise before the target in Experiment 2, but with an F0 rise in Experiment 3.

Participants accepted the lax target (with a preceding F0 rise) as intended, with 93.3% of the clicks on the lax target (upon hearing the lax phonetic form) in the non-tensifying “/N/+lax” condition, with 43.7% of the clicks on the lax target (upon hearing the derived tense form) in the tensifying “/k/+lax” condition, and with just 14.1% of the clicks on the lax target (upon hearing the phonetic tense form) in the non-tensifying “/N/+tense” condition (see Fig 7). As in Experiment 2, the analysis made use of a generalized linear mixed effect model with a binomial linking function with the tensifying “/k/+lax” condition mapped on the intercept and two dummy-coded variables for the non-tensifying “/N/+lax” and “/N/+tense”conditions. Subject and item were used as random factors with random slopes for both dummy variables. The results showed significantly more clicks on the lax target, as expected, in the “/N/+lax” than in the tensifying “/k/+lax” condition (B = 5.092 (0.727), z = 6.916, *p* < 0.001) and more clicks on the lax target in the tensifying “/k/+lax” condition than in the “/N/+tense” condition (b = -2.370 (0.371), z = -6.397, *p* < 0.001).

A mixed effect model was also used to compare the current results with those of Experiment 2 by adding the predictor Experiment as a numerical contrast (Experiment 2: -0.5, Experiment 3: 0.5). As shown in Table 5, there was a significant effect of Experiment, which indicates a significant difference between the two experiments for the intercept (the tensifying “/k/+lax”) condition (rather than the main effect of Experiment). In other words, the significant effect of Experiment suggests that participants are less likely to accept the lax word as the target upon hearing a derived tense phonetic form in the tensifying “/k/+lax” condition with an F0 rise (Experiment 3) than without an F0 rise (Experiment 2).

**Table 5:** Results of the cross-experiment comparison for click responses in Experiments 2 and 3 with the intercept mapped on the “/k/+lax” condition.

Effect	b	Z	p
Intercept	-0.169 (0.279)	-0.606	0.545
“/N/+lax”	6.873 (0.929)	7.398	< .001
“/N/+tense”	-2.653 (0.271)	-9.799	< .001
Experiment	-0.363 (0.149)	-2.432	0.015
“/N/+lax” x Experiment	-3.862 (1.085)	-3.559	< .001
“/N/+tense” x Experiment	1.106 (0.278)	3.980	< .001

The effect of Experiment, however, was not simply because participants in Experiment 3 were less likely to accept the lax words as targets across the board. There was also a significant interaction of Experiment with the “/N/+tense” predictor (the bottom line in Table 5), indicating that the opposite was true in this condition, as shown in Fig 7C. It appears that the more clicks on the lax target in “/N/+tense” in Experiment 3 than in Experiment 2 is at least partly due to the possibility that the preceding F0 rise leads to a relatively lower F0 percept associated with the following tense target. Given that one of the important cues to the tense stop is a higher F0 at the onset of the vowel and a lower F0 for the lax stop in Korean [28], the relatively lowered F0 percept of the following vowel would give rise to more looks to the lax target than when there was no such relative lowering of the F0 in Experiment 2. The critical comparison was therefore to what extent participants would accept the lax target upon hearing a derived tense form, as in Fig 7B, depending on the preceding prosodic (F0) context (Experiment 2 vs. Experiment 3). The statistical results indeed indicated that listeners looked significantly *less* to the lax target when preceded by an F0 rise (Experiment 3) than when there was no F0 cue.

**Fig 8:**
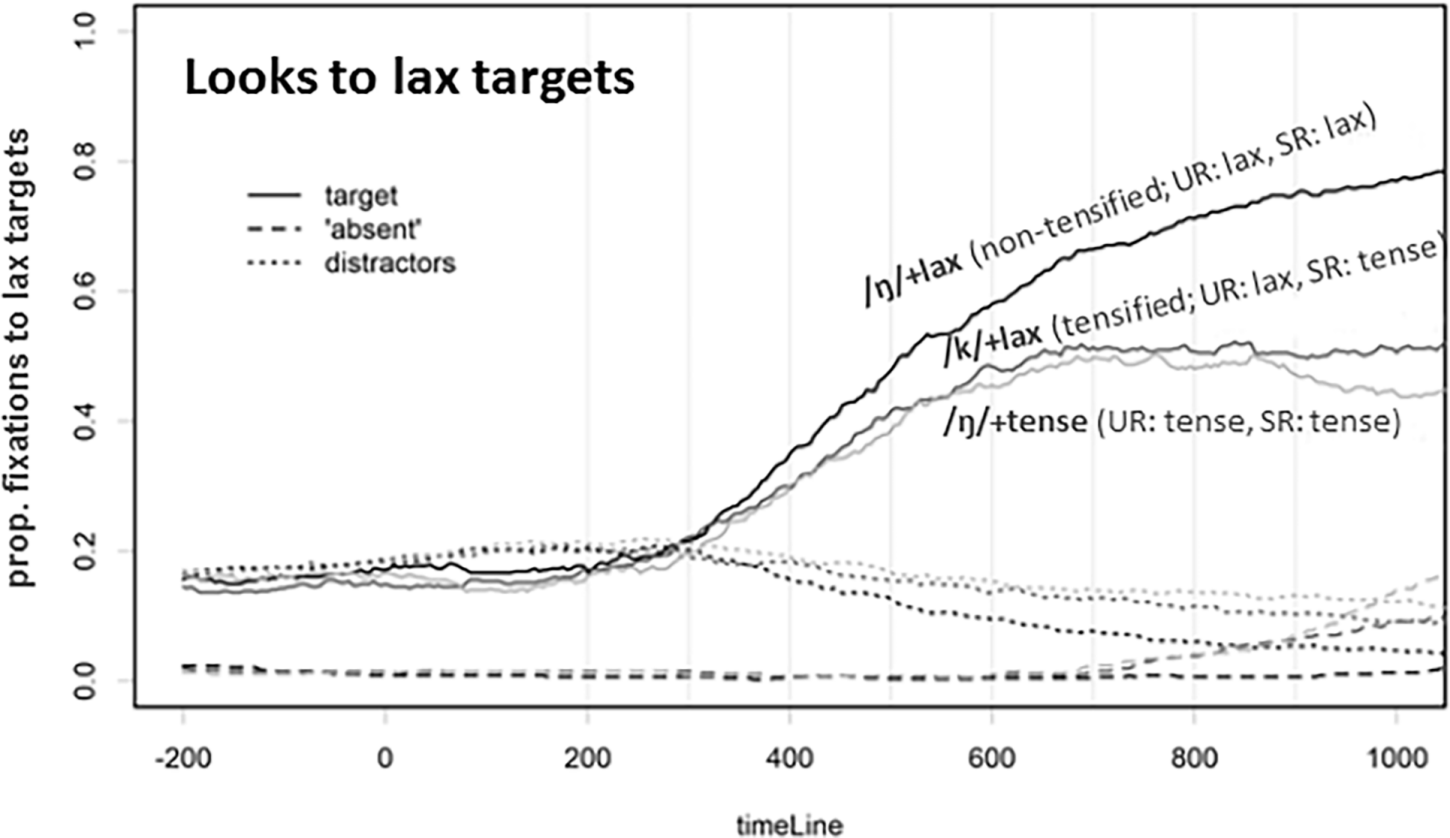
Fixations on the lax target, the “absent” symbol, and the distractors in Experiment 3 (with the phrase-final F0 rising cue in the precursor). The line type indicates object type (target vs. absent vs. distractors), and the critical comparison is between /k/+lax (tensified; Underlying Representation, UR = lax; Surface Representation, SR = tense) and /ŋ/+lax (non-tensified; Underlying Representation, UR = tense; Surface Representation, SR = tense).

Fig 8 shows the eye-tracking data for Experiment 3. In contrast to Experiment 2, there was no clear separation of the “/N/+tense” and the tensified “/k/+lax” conditions. The statistical analysis using 100ms time windows (see Table 6) revealed no clear difference between these two conditions, despite the clear difference in the number of clicks on the lax target, as illustrated in Fig 7. However, a cross-experiment comparison shows that there is a significant interaction with Experiment only in later time windows, starting from 800ms onward (see the rightmost column of Table 7 for the “/N/+lax” x Experiment interaction). This indicates that the influence of F0 that led to the convergence between the “/k/+lax” and the “/N/+tense” conditions in Experiment 3 became effective after 800ms.

**Table 6:** Results from the moving time window analysis from Experiment 3. The intercept was mapped on the “/k/+lax” condition.

time window	/N/+lax			/N/+tense		
	b (SE)	t	p	b (SE)	t	p
200–300	0.021 (0.143)	0.144	0.886	-0.092 (0.154)	-0.600	0.552
300–400	0.099 (0.162)	0.612	0.544	-0.096 (0.158)	-0.610	0.544
400–500	0.354 (0.196)	1.805	0.081	-0.062 (0.194)	-0.320	0.751
500–600	0.535 (0.183)	2.923	0.004	-0.136 (0.183)	-0.744	0.457
600–700	0.792 (0.186)	4.263	< .001	-0.144 (0.186)	-0.773	0.440
700–800	1.120 (0.234)	4.790	< .001	-0.157 (0.189)	-0.830	0.409
800–900	1.475 (0.280)	5.263	< .001	-0.126 (0.189)	-0.664	0.509
900–1000	1.635 (0.265)	6.179	< .001	-0.324 (0.190)	-1.707	0.100

**Table 7:** Cross-experiment comparison for the time course of looks to the lax targets. Note that the first column indicates the difference on the intercept condition, which was the tensifying “/k/+lax”.

time window	Experiment (on Intercept)		“/N/+lax” x Experiment		“/N/+tense” x Experiment	
	B (SE)	t	B (SE)	t	B (SE)	t
200–300	0.857 (0.162)	5.28***	-0.087 (0.228)	-0.381	-0.318 (0.228)	-1.4
300–400	0.971 (0.177)	5.49***	0.034 (0.241)	0.139	-0.331 (0.242)	-1.37
400–500	0.905 (0.182)	4.96***	0.270 (0.264)	1.02	-0.244 (0.258)	-0.944
500–600	0.79 (0.185)	4.27***	0.484 (0.262)	1.85.	-0.114 (0.282)	-0.405
600–700	0.536 (0.186)	2.88**	0.642 (0.262)	2.45**	0.052 (0.286)	0.182
700–800	0.546 (0.185)	2.95**	0.396 (0.262)	1.51	-0.192 (0.272)	-0.707
800–900	0.682 (0.184)	3.71***	0.084 (0.257)	0.325	-0.595 (0.257)	-2.31**
900–1000	0.752 (0.18)	4.17***	-0.098 (0.255)	-0.383	-0.652 (0.258)	-2.53**

(** refers to p<0.01, and *** to p<0.001)

Before moving to the discussion section, it is worth nothing that the significant effects of Experiment in the cross-experiment comparison (as given in the leftmost column of Table 7) are difficult to interpret because the exact time course differs between Experiment 2 and Experiment 3. In other words, comparing the results of Experiments 2 and 3 shows that looks to the target generally come earlier in Experiment 2 than in Experiment 3. (This might be due to a general slow-down caused by the PSOLA manipulation in the preceding context, which may have led participants to be more cautious in fixating a potential target.) Furthermore, the interaction effect for the “/N/+lax” x Experiment interaction, given in the middle column of Table 7, indicates that there is a more robust separation between the “/N/+lax” and “/k/+lax” conditions in Experiment 3 at 600ms, which also appears to be interpretable in connection with the critical F0 condition present in Experiment 3.

### 4.3 Discussion

Results showed that, upon hearing a tense form, participants clicked on the lax target significantly less often in Experiment 3 in the presence of an F0 cue to a prosodic boundary in the preceding context than in Experiment 2 with no such F0 cue present in the same position. In addition, unlike Experiment 2 in which listeners showed a phonological inferencing effect (with more looks to the lax target in the tensifying context than in the non-tensifying context), the effect was not observed in Experiment 3. That is, there was no difference in their looks to the lax target regardless of whether they heard a derived tense form (in the tensifying (“/k/+lax”) condition) or an underlying tense form (in the non-tensifying (“/N/+tense”) condition). The results strongly suggest that prosodic boundary information modulates phonological inference. In this specific case of Korean, participants did not show evidence of phonological inferencing when there was an F0 rise cue for an AP boundary between the preceding rule-triggering context and the target word. The AP boundary was cued in the signal only by a high F0 without any temporal adjustment such as phrase-final lengthening or a pause. This suggests that an F0 cue by itself suffices to signal a prosodic boundary as long as it is specified as such in the prosodic grammar of a language, as is the case with an AP in Korean [10, 27].

The question follows: when does prosodic information start exercising its influence on segmental processing? In Experiment 2, there was an early effect of phonological inferencing; however, the comparison of the time course of eye movement to the lax targets between Experiments 2 and 3 indicated no significant between-experiment effect in early time windows. Statistical analyses between the two experiments across different time windows suggested that the effect of prosodic structure on phonological inferencing comes into effect relatively late in the process. Only after some time elapsed (i.e., after 800ms) did the prosodic information have an effect, so that the difference between the tensifying (“/k/+lax”) condition and the non-tensifying (“/ŋ/+tense”) condition found in the later windows in Experiment 2 was not observed in Experiment 3. It is worth noting, however, that there was a non-significant numeric trend toward an early effect of prosodic influence, leaving the possibility that an early effect may still exist, although not as strongly as the observed late effect.

Before we move on to the general discussion section, a word of caution is in order about a possible artifact that may have arisen due to the nature of the task involved in Experiments 2 and 3. Recall that we found evidence for phonological inference in retrieving the underlying lax stop in the absence of the tense competitor on the screen which was replaced with the “absent” option. As a reviewer pointed out, one might not rule out a possibility that the more clicks on the lax target than on the “absent” option were due to the fact that the lax target was accompanied by a shape but the “absent” option was not, resulting in a bias towards the lax target. We, however, believe that the ‘click’ data can still be interpretable in terms of phonological inference for the following reasons. First, listeners did click on the “absent” option about 91% of the time in the non-tensifying context, despite the lack of the shape associated with the “absent” option. Second, the evidence for phonological inference obtained with the ‘click’ data was also reflected in the on-line ‘look’ data which is supposed to be influenced minimally by the shape information. Third, there was a difference in the ‘click’ data between Experiments 2 and 3, which made the results interpretable in terms of prosodic modulation of phonological inference despite the fact that both experiments contained the “absent” option with no shape information.

## General discussion

Three eye-tracking experiments explored to what extent listeners would recover the underlying form of a segment altered by a phonological rule application during the time course of spoken language processing and how and when prosodic structure information come into effect, further modulating such context-dependent phonological inferencing. We addressed these questions with a case of the post-obstruent tensing (POT) rule in Korean, by which an underlying lax (lenis) consonant is categorically tensified (becoming a fortis consonant) when it follows an obstruent within an Accentual Phrase (AP). An important characteristic of AP in Korean is that, unlike other prosodic phrases that mostly involve temporal cues (e.g., final lengthening) along with phrase boundary tones at the edges, the Korean AP may be formed primarily by a phase-edge tone without substantial temporal cues [30]. This allowed us to further examine whether listeners’ segmental decisions can be attributable primarily to their computation of prosodic structure itself, rather than to their adjustment of perception according to a change in local speech rate reflected in the low-level temporal dimension of the signal, as has been discussed in [14] and [16].

Experiment 1 was carried out as a baseline experiment to confirm whether a potential ambiguity arising with a phonetically tense form in a phrase-internal context (which may be either a derived tense due to POT or an underlying tense form) would categorically disappear at the strongest prosodic juncture (i.e., across an Intonational Phrase boundary, which is expected to block the POT rule completely). Listeners indeed showed the difference between the two boundary conditions (IP vs. Word), with more looks to the intended targets in an IP than in a word boundary. In the word boundary condition, however, upon hearing a derived tense form (from an underlying lax), listeners did not look at the lax target at all until they were told to do so by the instruction in the carrier sentence. (Recall that the ambiguity was eventually disambiguated by telling the participant to click on a shape above the intended target.) In other words, listeners preferred to look at the tense target that was auditorily faithful to the phonetic input (i.e., a derived tense form from a lax stop) in the speech signal. Given that the application of POT leads to a categorical segmental change within the prosodic domain of AP [10, 27], and that the results of our pretest showed that the listeners treated both the derived and the underlying tense forms in isolation equally as tense, the null effect was interpreted as being due to the listeners’ propensity to prioritize the acoustic-auditory match between the auditory input and the target as long as the phonetically faithful tense target appears on the visual display. It would then make it difficult to observe the effect of phonological inferencing that, in principle, would otherwise have been observed (e.g., [21–23]). This is in line with observations that listeners tend to prefer interpretation in which the realization of a segment is faithful to its underlying form rather than assuming a contextual influence. For example, gating studies of nasalization have shown that listeners from languages with an opposition of nasalized and oral vowels prefer to interpret a nasalized vowel as underlyingly nasal rather than nasalized due to an adjacent nasal consonant [42–44]. In the subsequent experiments, therefore, we adapted the visual display with the lax option and an “absent” symbol instead of the tense option, which listeners could click on if they thought there was no word on the screen that matched the auditory stimulus.

Experiment 2 was conducted with the new visual display and with only the word boundary context to test if the phonological inferencing effect could be observed in the rule-triggering prosodic context. Results showed that listeners indeed accepted a lax target as a potential match upon hearing a derived tense form, and that they gave more looks to the lax target when they heard a derived tense form in the tensifying context than when they heard the underlying tense form in the non-tensifying context, in earlier and later time windows (although there were time periods in the middle in which their looks did not differ in the two conditions). Recall that, in Experiment 1, listeners looked to the tense target for an auditory input of both a derived and an underlying tense form. These results thus provide strong evidence for phonological inferencing even in the case of categorical neutralization. This is, listeners undo the phonological rule in the phonologically legitimate context (in this case, a tensifying context across a phrase-internal word boundary).

With these results, we further tested in Experiment 3 whether, and if so how, an F0 cue without any other cues to the prosodic juncture is employed by the listeners in computing prosodic structure and modulating lexical access. Only F0 of the preceding context word was manipulated from the same stimuli used for Experiment 2 with all the other acoustic information intact, such that the stimuli had a high F0 in the end of the preboundary position, consistent with the AP-final edge tone in Korean. Contrary to the findings of Experiment 2, listeners did not show any difference in processing the derived versus the underlying tense forms, suggesting that the presence of an F0 cue to a prosodic phrase boundary (in this case an AP boundary) blocked the phonological inferencing process.

As the prosodic boundary cue was given only by a heightened F0 value without any temporal cues in the current study, the results enable us to draw a firmer conclusion that it is not the low-level speech rate normalization but the higher-level linguistic structure (i.e., the prosodic boundary *per se*) that influences listeners’ segmental decisions. The results of the present study build on a gradually increasing body of literature on the role of language-specific prosodic cues on speech processing. For example, an F0 rise may be an important cue to the onset of a word in English, especially given the statistics of prominence distribution across words [45], but an F0 rise may be used to signal the end of a word in French or Korean, as a word in these languages often ends with an F0 rise in conjunction with an intonational phonology [10, 27, 46]. Likewise, the effect of F0 rise found in the present study suggests that the language-specific F0 pattern as stipulated by the grammar of the sound system of a given language is indeed used by the listener in speech comprehension. That said, the findings of earlier studies that have shown some degree of prosodic modulation to speech categorization and phonological rule processing [14–16] could be explained in a similar vein.

However, we do not undermine the role of universally applicable temporal adjustment that appears at the edges of prosodic constituents, as their critical role in speech processing has long been attested to in numerous studies [47, 48]. Rather, the current study suggests that listeners can compute prosodic structure independently of temporal adjustment, and that the suprasegmental cues to the phrase boundary, be it temporal or spectral, contribute to the computation of higher-level linguistic prosodic structure, which is hypothesized to modulate speech perception and spoken word recognition (e.g., [13, 17, 18]).

The current study also provided us with an opportunity to observe the time course of prosodic modulation by comparing Experiments 2 and 3—i.e., in the absence and presence of an F0 cue, respectively. Results suggest that prosodic modulation of the phonological inferencing process comes into full effect relatively late in the time course of speech perception. This supports the claims that suprasegmental information is processed in a different time scale from, but integrated later with, segmental information [30] or that prosodic structure is parsed in parallel to segmental level and is used later for prosodic modulation in lexical access [13].

In conclusion, the results taken together suggest that an acoustically identical input for the target is processed with differential degrees of lexical activation over the time course depending on the computed prosodic structure. An F0 cue alone can be exploited in this computation process, fine-tuning lexical processing through phonological inferencing in a language-specific way. This also implies the importance of understanding speech processing in conjunction with prosodic structural analysis, warranting further research into the role of language-specific vs. universally applicable prosodic cues in speech comprehension.
